# The Heating and Lighting of Hospital Wards

**Published:** 1909-03-06

**Authors:** 


### HOSPITAL ADMINISTRATION.

### CONSTRUCTION AND ECONOMICS.

### THE HEATING AND LIGHTING OF HOSPITAL WARDS.

V.—METHODS OF LIGHTING.

The tendency of artificial lighting during the last fifteen to twenty years has been in the direction of larger individual sources of light. The Welsbach burner is claimed, for instance, to give 60 candles, for a consumption of less gas than was able to produce 12 to 14 candles in the old open burner. And other forms of artificial light have followed in the same direction, partly because there has been a continual tendency to require more light since the first advent of the electric arc lamp, and partly because the advantages of the different improvements that have been made in gas, electricity, and petroleum lights are all more easily obtained in large forms than in small. It may be taken as a rule, also, that large individual lights are produced and maintained more economically than small ones. For comparatively large rooms, such as modern hospital wards, with fairly high ceilings, a good general illumination can be obtained by the use of a small number of large lamps in place of, as at present is usual, a large number of small lamps. Electric incandescent lamps of large power, up to 500 candles, are made of thoroughly reliable form and of very much greater efficiency than the ordinary carbon-filament lamp, and for the ordinary supply pressures that are common in town services. Gas lamps formed of a group of inverted mantles are also on the market; they are of large powers, and have been worked out to rival the incandescent and arc electric lamps, and they are also thoroughly satisfactory, subject to the remarks made below, about gas lighting generally. A few large electric incandescent electric lamps or large inverted mantle gas lamps, suspended well above the patient's heads, would produce a very pleasant illumination, very much better and more generally useful than the present system of small lamps. For special work, such as dressing patients at night, etc., where the electric light is used, plug connections can be easily, and at small cost, placed between the beds, and one or two portable lamps can be attached to them, by the nurse on duty, as they are required. In addition to general economy, this arrangement has the advantage of concentrating the light upon the bed required, and of not being an annoyance to the patients in adjoining beds.

#### Sources of Light Available.

For town hospitals the two most economical and most convenient sources are the town gas supply and the town electricity supply. It is thoroughly practicable for any hospital to have its own gasworks and its own electricity generating station; but in towns it will be found far more satisfactory in every way to take a supply from the town service. The keen competition between gas and electricity has, for the present at any rate, put private generating stations within the town radius of supply out of court. For country hospitals, sanatoria, convalescent homes, etc., that are out of the reach of the town supply of electricity or gas, the sources of light available are petroleum lamps, petroleum incandescent lamps, the new so-called air-gas, acetylene gas, and small gasworks, or small electricity generating stations.

The question of the relative cost of the different lights will be dealt with in the next article. With regard to the relative advantages, there is not much to choose between gas and electricity, so far as convenience is concerned, with modern developments. Gas engineers have closely followed electricians; and by the aid of the pilot light and pneumatic tubes, and even auxiliary electrical apparatus, gas lamps, such as those suggested for suspension overhead in the wards, can be turned in and out from any convenient point, such as the nurse's room, just as easily as electric lights can. In appearance also it is difficult to imagine anything more pleasing to the eye or producing a nicer effect than the latest forms of inverted mantle, shaded with ground glass or opal globes. On the other hand, gas has the distinct disadvantage that it is never all consumed whether used for lighting or heating, except in a very few modern heating appliances, and consequently there is a continual passage out into the ward of a small quantity of the unburned gas itself, besides the products of combustion. According to the latest researches on the subject it is not the small quantity of carbonic acid that is so harmful, but the gas itself which has not been completely burned. The same thing applies, in a smaller degree, to petroleum and petroleum incandescent gas. In addition, with these lamps, and with any apparatus using petroleum, there is sometimes the unpleasant smell with which everyone is familiar. This may be due to two things—badly-made apparatus or improper attendance. The petroleum does not leak through the receptacle containing it, but works over from the reservoir by capillary action, and if the apparatus is properly designed and kept clean there should be no danger of this. It is fair to say that modern lamps are thoroughly satisfactory in this matter, so far as construction is concerned. Acetylene gas is easily made, but it is open to even graver objection than ordinary coal gas, because of the nature of the gas itself. Further, great care has to be exercised in the matter of generation to avoid possible explosions. This, however, is again a matter of care. The apparatus on the market are all exceedingly simple and easily understood.

The electric light hardly needs any explanation. Air gas is merely air charged with 1½ per cent. of the vapour of petrol. It is consumed in specially prepared mantles.

